# Low *Ten-eleven-translocation 2* (*TET2*) transcript level is independent of *TET2* mutation in patients with myeloid neoplasms

**DOI:** 10.1186/s13000-016-0476-4

**Published:** 2016-03-16

**Authors:** Renata Scopim-Ribeiro, João Agostinho Machado-Neto, Paula de Melo Campos, Fernanda Soares Niemann, Irene Lorand-Metze, Fernando Ferreira Costa, Sara Teresinha Olalla Saad, Fabiola Traina

**Affiliations:** Hematology and Hemotherapy Center, University of Campinas/Hemocentro-Unicamp, Instituto Nacional de Ciência e Tecnologia do Sangue, Rua Carlos Chagas, 480, CEP 13083-878, Campinas, SP Brazil; Present address: Department of Internal Medicine, University of São Paulo at Ribeirão Preto Medical School, Ribeirão Preto, SP Brazil

**Keywords:** Ten-eleven-translocation 2, TET2, Mutation, Myelodysplastic syndromes, Acute myeloid leukemia

## Abstract

**Background:**

New sequencing technologies have enabled the identification of mutations in *Ten-eleven-translocation 2* (*TET2*), an enzyme that catalyzes the conversion of 5-methylcytosine into 5-hydroxymethylcytosine (5-hmC) in myeloid neoplasms. We have recently identified reduced *TET2* mRNA expression in myelodysplastic syndromes (MDS) and acute myeloid leukemia (AML), which is associated with a poor overall survival in MDS. We herein aimed to investigate *TET2* mutations and their impact on *TET2* expression in a cohort of patients with myeloid neoplasms, including MDS and AML patients.

**Findings:**

*TET2* mutations were observed in 8 out of 19 patients (42 %) with myeloid neoplasms. The *TET2* expression profile was similar between in wild type and in *TET2* mutated patients.

**Conclusion:**

Our results suggest that *TET2* expression is reduced in MDS/AML patients, independently of mutational status.

## Findings

### Introduction

New sequencing technologies have enabled the identification of several mutations in epigenetic regulator genes, such as *Ten-eleven-translocation 2* (*TET2*), which encodes an enzyme that catalyzes the conversion of 5-methylcytosine into 5-hydroxymethylcytosine (5-hmC). Mutations in the coding regions of *TET2* lead to loss of TET2 protein function, reduce 5-hmc levels and cause a significant shift towards monocyte/granulocyte differentiation in detriment of erythroid/lymphoid differentiation [1, 2]. *TET2* mutations confer a worse prognosis in myelodysplastic syndromes (MDS) and acute myeloid leukemia (AML) [3, 4]. We have recently reported that *TET2* mRNA expression is downregulated in MDS and AML, and predicts poor overall survival in MDS patients [5]. However, the correlation between the presence of *TET2* mutations and *TET2* transcript levels has rarely been addressed [4, 6]. We, herein, expanded our previous observations and aimed to investigate *TET2* mutational status and the impact of this mutational status on *TET2* expression in a cohort of MDS and AML patients. For this purpose, *TET2* mutation analysis and *TET2* gene expression were tested by Sanger sequencing and quantitative PCR, respectively, in bone marrow samples from healthy donors and myeloid neoplasm patients.

### Materials and methods

#### Patients’ characteristics

Bone marrow samples were obtained from a total of 22 healthy donors, and 19 patients with myeloid neoplasms (MDS = 10 and AML = 9) followed at the outpatient clinics of the University of Campinas, who had also been included in a previous study [5]. The present study was approved by the Institutional and National Review Board in accordance with the Helsinki Declaration. Written informed consent was obtained from all patients who participated in this study. Patients were diagnosed according to the 2008 Word Health Organization criteria [7]. Patients’ characteristics are described in Table 1.

**Table 1:** Patients’ characteristics

Patients	Number
MDS	10
Gender	
Male/Female	5/5
Age (years), median (range):	58 (28–78)
WHO 2008	
RA	2
RARS	1
RCMD	4
RAEB-1	3
IPSS-R	
Low	5
Intermediate	2
High	1
Not available	2
Cytogenetics	
Normal karyotype	10
BM blast (%)	
<5 %	7
≥5 and <10 %	3
AML	9
Gender	
Male/Female	4/5
Age (years), median (range)	66 (44–90)
BM blasts (%), median (range)	92 (31–98.5)
Cytogenetics	
Normal karyotype	2
Deletion 5q	1
Monosomy 7	1
Complex karyotype	1
No growth	4

Abbreviations: *MDS* myelodysplastic syndromes, *WHO* World Health Organization, *RA* refractory anemia, *RARS* refractory anemia with ringed sideroblasts, *del(5q)* MDS with isolated del(5q), *RCMD* refractory cytopenia with multilineage dysplasia, *RAEB-1* refractory anemia with excess blast-1, *BM* bone marrow, *IPSS-R* Revised International Prognostic Scoring System, *AML* acute myeloid leukemia

#### Polymerase chain reaction (PCR) and DNA sequencing

The screening of *TET2* mutations was performed on coding exons 3, 4, 5, 6, 7, 8, 9, 10 and 11 (GenBank reference NM_0175628.4). Primer sequences and PCR conditions were previously described [8]. Amplicons were sequenced with an ABI 3500 Genetic Analyzer (Life Technologies, Carlsbad, CA, USA) using the Big Dye terminator V1.1 cycle sequencing kit and analyzed using CLC Main Workblench v.7.6.2 software (Qiagen, Aarhus, Denmark). All alterations found were searched in SNP (dbSNP; http://www.ncbi.nlm.nih.gov/projects/SNP), Ensembl Genome Browser databases (http://asia.ensembl.org/index.html), and in the Catalogue of Somatic Mutations in Cancer (COSMIC; http://cancer.sanger.ac.uk/cosmic).

#### Quantitative PCR (qPCR)

Total RNA was extracted from cells using the TRIzol reagent (Life Technologies, Carlsbad, CA, USA). The reverse transcription reaction was performed using RevertAid™ First Strand cDNA Synthesis Kit (MBI Fermentas, St. Leon-Rot, Germany). *TET2* mRNA level was detected by Maxima Sybr green qPCR master mix (MBI Fermentas, St. Leon-Rot, Germany) in the ABI 7500 Sequence Detection System (Life Technologies) using specific primers: forward 5′- ACGCAAGCCAGGCTAAACA -3′, reverse 5′- GCTGGGACTGCTGCATGA -3′; *HPRT1* (*hypoxanthine phosphoribosyltransferase 1*) was used as the endogenous control: forward 5′-GAACGTCTTGCTCGAGATGTGA-3′, reverse 5′-TCCAGCAGGTCAGCAAAGAAT-3′. The relative quantification value was calculated using the equation, 2^-ΔΔCT^ [9]. A negative ‘No Template Control’ was included for each primer pair. The dissociation protocol was performed at the end of each run to check for non-specific amplifications. Three replicas were run on the same plate for each sample.

#### Statistical analyses

Statistical analyses were performed using GraphPad Instat 5 (GraphPad Software, Inc., San. Diego, CA, USA). For comparisons, Mann-Whitney test was used for measured factors with two levels. A *p* value <0.05 was considered as statistically significant.

### Results and discussion

**Fig. 1:**
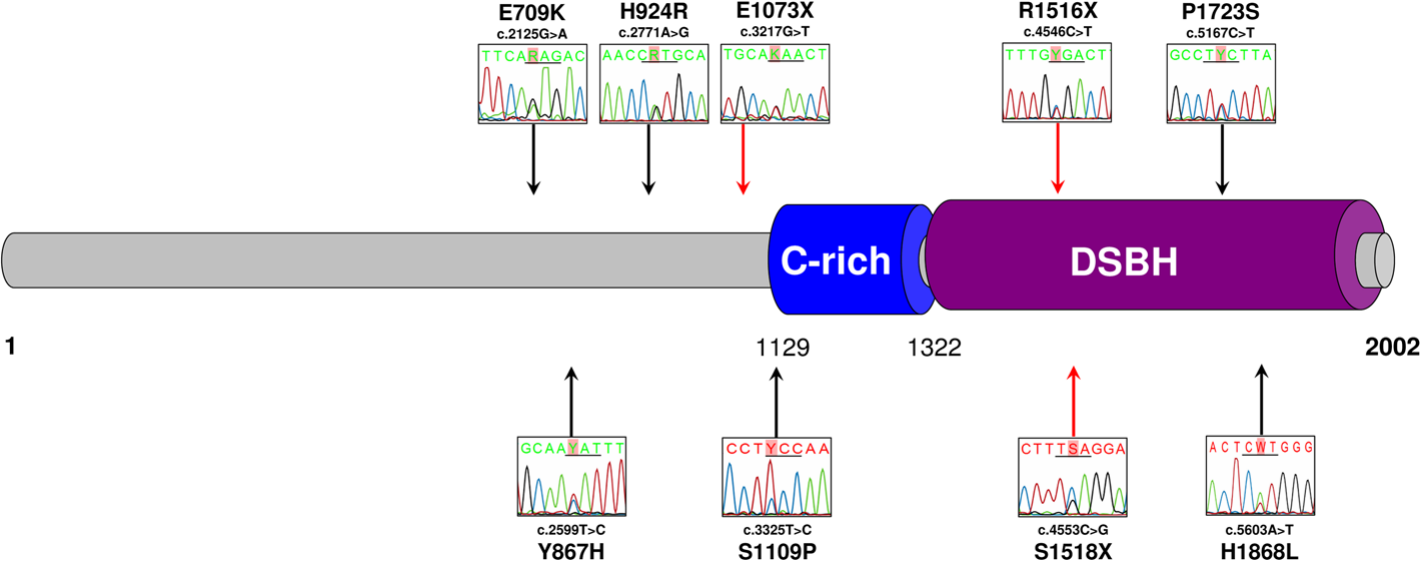
*TET2* mutations identified in myelodysplastic syndromes and acute myeloid leukemia patients. In a cohort of 19 patients, nine *TET2* mutations were identified in eight patients. Genomic sequencing of protein-coding regions revealed missense (*black arrows*), and stop codon (*red arrows*) mutations in *TET2*; Sanger sequencing analysis is illustrated in the figure. TET2 protein primary structure indicating the domains and specific known conserved motifs are shown: cysteine-rich region (C-rich), double strand beta helix (DSBH). The aminoacid position is indicated

We analyzed the mutation status of *TET2* exons and the impact of this status on *TET2* expression in bone marrow cells from myeloid neoplasm patients. In total, 17 *TET2* variants were detected. After excluding confirmed SNPs (P29R [rs12498609], V218M [rs6843141], P363L [rs17253672], G355D [rs61744960], H1778R [rs62621450], I1762V [rs2454206] and L1721W [rs34402524]), *TET2* mutations were observed in 8/19 (42 %) patients with myeloid neoplasms [4/10 (40 %) MDS and 4/9 (44 %) AML], including six missense (E709K, Y867H, H924R, S1109P, P1723S and H1868L) and three stop codon (E1073X, S1516X and S1518X) mutations (Fig. 1). A total of nine *TET2* mutations were found in exon 3 (*n* = 5) or exon 11 (*n* = 3) in eight patients; one patient had two mutations. Four *TET2* mutations (E1073X, S1109P, S1518X and H1868L) found in our study had not been previously described in the COSMIC database. Among the six missense mutations, three mutations presented a high probably of damage to protein function, according to Polyphen2 analysis. The PolyPhen2 scores for predicted damage of TET2 Y867H, S1109P and H1868L mutations were 0.999, 0.995 and 1, respectively (http://genetics.bwh.harvard.edu/pph2/). *TET2* mutations in exons have been implicated in protein loss-of-function and myeloid neoplasm mechanism of disease [1, 2], which supports our focus on sequencing *TET2* coding regions only. The relevance of mutations in the *TET2* promoter region has not been explored yet in myeloid neoplasms.

**Fig. 2:**
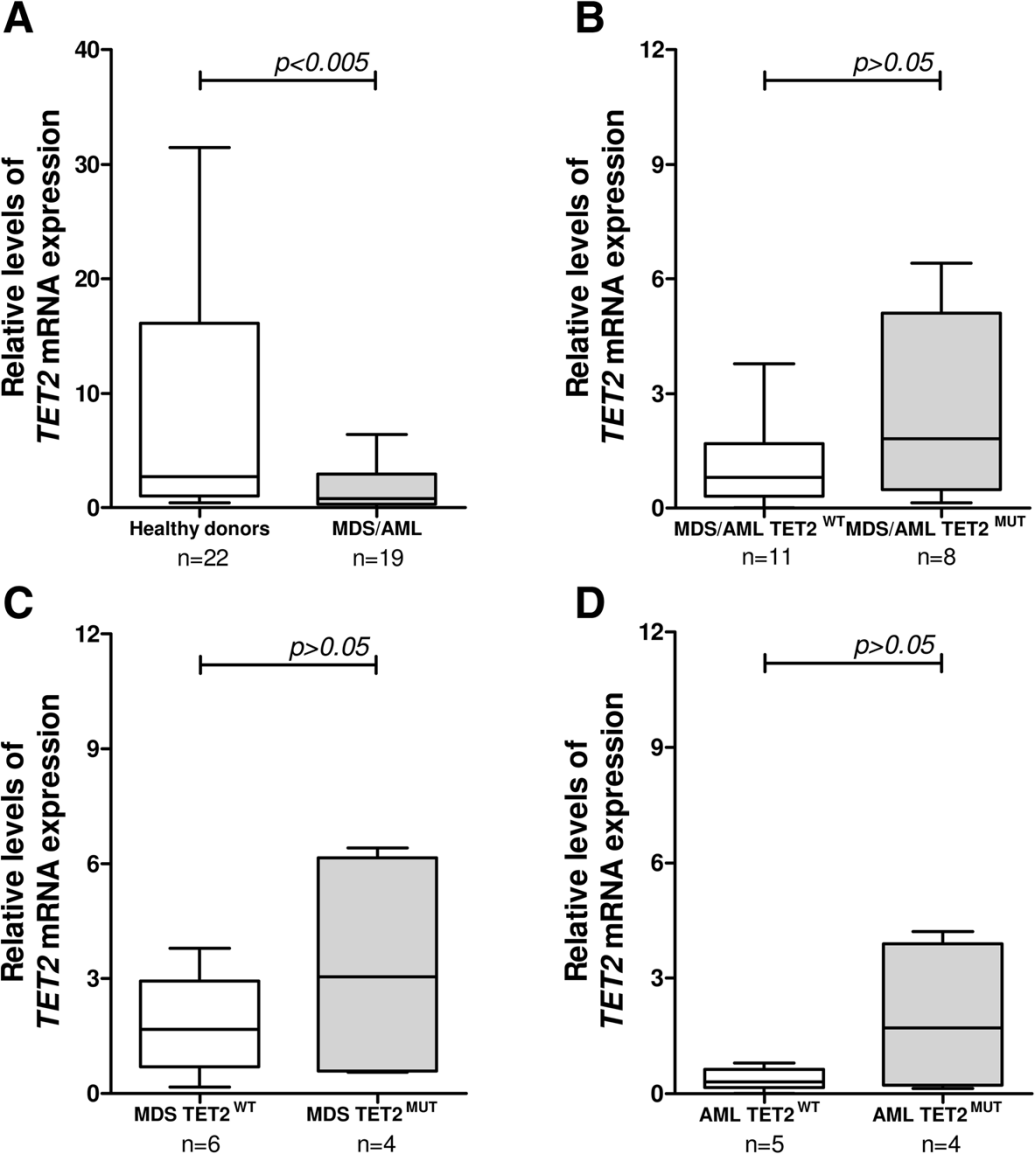
*TET2* expression, according to *TET2* mutational status, in myelodysplastic syndromes and acute myeloid leukemia patients. **a** qPCR analysis of *Ten-eleven-translocation 2 (TET2*) mRNA levels in total bone marrow cells from healthy donors and from patients with the diagnosis of myelodysplastic syndromes (MDS)/acute myeloid leukemia (AML). *TET2* expression in patients stratified according to *TET2* mutational status for the entire cohort of MDS/AML patients (**b**), for patients with MDS (**c**) or AML diagnosis (**d**). The “y” axis represents the relative *TET2* mRNA expression. The numbers of subjects studied and the *p* values (Mann-Whitney) are indicated in the graph. Abbreviations: *WT* wild type, *MUT* mutated

*TET2* expression was reduced in MDS/AML patients (median 0.8 [minimum 0.01- maximum 6.41]), compared with healthy donors (2.72 [0.43–31.49]); *p* <0.005 (Fig. 2a). The standard deviation of *TET2* expression for the healthy donors was high in our cohort of patients, which is in agreement with reports from other authors [4, 10]. *TET2* expression was similar in *TET2* wild type (0.8 [0.01–3.79]) and in mutated patients (1.82 [0.14–6.41]), for the entire cohort, and among patients with MDS and AML only, all *p* >0.05 (Fig. 2b–d). These findings are in agreement with Jankowska and colleagues [4] and Coutinho and colleagues [6], who reported *TET2* downregulation in 16 patients with MDS/myeloproliferative neoplasms and in 12 patients with pediatric MDS, respectively, regardless of *TET2* mutational status. Reduced *TET2* levels in MDS patients have also been previously reported by our group [5], Li and collaborators [10], and Zhang and collaborators [11]. However, the *TET2* mutation was not evaluated in these studies.

Somatic mutations in *TET2* have been described in healthy elderly individuals with clonal hematopoiesis and have been associated with alterations in DNA methylation [12]. *TET2* mutations have been indicated as early events in clonal evolution and potently lead to the development of preleukemic hematopoietic stem cells [13, 14]. For this reason, growing attention has been given to the hypothesis that *TET2* is a gate keeper in hematological malignancies. The occurrence of *TET2* mutations may indirectly lead to the disruption of other important genes by inducing epigenetic changes, as recently described for *JAK2* [15]. *TET2* mutations alter the transcriptional consequences of JAK2 V617F in a cell-intrinsic manner and prevent JAK2 V617F from up-regulating a proliferative program [15]. In addition, Kameda et al. [16] and Rasmussen and collaborators [17] demonstrated, using mouse models, that the loss of Tet2 contributes towards the progression of myeloid neoplasms. Thus, reduced *TET2* function, due to loss-of-function mutations, reduced mRNA expression due to epigenetic silencing or not yet elucidated mechanisms may have a great contribution to the malignant phenotype of hematological cancers.

Taken together, our findings suggest that decreased *TET2* expression is observed in myeloid neoplasms and does not correlate with *TET2* mutation status. Other molecular mechanisms, including mutations of additional genes and/or epigenetic regulation may affect *TET2* expression in myeloid neoplasms [18]. *TET2* mRNA expression levels, in addition to *TET2* mutation status, may also play a role in myeloid neoplasm physiopathology. Future studies to further investigate the mechanisms that lead to reduced *TET2* expression in hematological malignancies will be of importance.

## Abbreviations

5-hmC: 5-hydroxymethylcytosine
AML: acute myeloid leukemia
COSMIC: catalogue of somatic mutations in cancer
HPRT1: hypoxanthine phosphoribosyltransferase 1
JAK2: Janus kinase 2
MDS: myelodysplastic syndromes
PCR: polymerase chain reaction
qPCR: quantitative PCR
SNPs: single nucleotide polymorphisms
TET2: ten-eleven-translocation 2
